# Genome-Wide Association Analysis and Breeding-Oriented SNP Marker Development for Bacterial Wilt Resistance in Tomato (*Solanum lycopersicum* L.)

**DOI:** 10.3390/plants14193036

**Published:** 2025-10-01

**Authors:** Anjana Bhunchoth, Wasin Poncheewin, Arweewut Yongsuwan, Jirawan Chiangta, Burin Thunnom, Wanchana Aesomnuk, Namthip Phironrit, Bencharong Phuangrat, Ratree Koohapitakthum, Rungnapa Deeto, Nuchnard Warin, Samart Wanchana, Siwaret Arikit, Orawan Chatchawankanphanich, Vinitchan Ruanjaichon

**Affiliations:** 1 National Center for Genetic Engineering and Biotechnology (BIOTEC), National Science and Technology Development Agency (NSTDA), Pathum Thani 12120, Thailand; 2 Department of Agronomy, Faculty of Agriculture at Kamphaeng Saen, Kasetsart University, Nakhon Pathom 73140, Thailand; 3 Rice Science Center, Kasetsart University, Nakhon Pathom 73140, Thailand

**Keywords:** tomatoes, bacterial wilt resistance, GWAS, SNP marker

## Abstract

Bacterial wilt, caused by *Ralstonia solanacearum*, is a major constraint to tomato production globally. To uncover resistance loci and develop efficient molecular tools for breeding, we conducted disease phenotyping over two growing seasons, which revealed consistent variation in resistance and moderate broad-sense heritability (H^2^ = 0.22–0.28), suggesting a genetic basis. A genome-wide association study (GWAS) was performed on a diverse panel of 267 tomato accessions, evaluated against two *R. solanacearum* strains. A major resistance locus was identified on chromosome 12, with the strongest association observed at SNP S12_2992992, located within a gene encoding a leucine-rich repeat (LRR) receptor-like protein. Haplotype analysis indicated that the resistance-associated allele is relatively rare (~13.5%) in the population, underscoring its potential value in breeding programs. Functional validation in an F_2_ population derived from a cross between the susceptible ‘Seedathip6’ and the resistant ‘Hawaii 7996’ confirmed that the TT genotype at S12_2992992 was significantly associated with enhanced resistance. A Kompetitive Allele Specific PCR (KASP) marker was developed for this SNP, facilitating cost-effective and high-throughput selection. Collectively, these findings establish S12_2992992 as a robust and functionally informative marker, offering a valuable tool for accelerating bacterial wilt resistance breeding in tomato through marker-assisted selection.

## 1. Introduction

Bacterial wilt (BW) disease, caused by *Ralstonia solanacearum*, represents one of the most widespread plant pathologies globally, with prevalence in the tropical and humid regions such as Thailand. This pathogen impacts the vascular tissue of a broad spectrum of crops, detrimentally affecting their productivity and posing a substantial threat to global agriculture [1,2,3]. As Gram-negative bacterium, *R. solanacearum* causes damage throughout all developmental stages of crop growth. The infection mechanism involves pathogen entry through natural plant openings, subsequent colonization of host tissues, and systemic dissemination throughout the plant. The bacterium produces excessive quantities of exopolysaccharide (EPS), which accumulates xylem vessels, resulting in vascular obstruction that leads to water stress, progressive wilting, and ultimately plant death [4,5]. *R. solanacearum* demonstrates remarkable environmental adaptability and persistence, enabling survival in various conditions including contaminated agricultural equipment. The taxonomic classification of this pathogen encompasses 5 distinct races and 6 biovars, reflecting its diverse host specificity and biochemical characteristics [6]. This diversity enables the pathogen to infect more than 200 plant species across 50 different botanical families [7]. The economic consequences of bacterial wilt disease are substantial, causing significant agricultural losses in both developing and developed nations [8,9]. Consequently, effective management strategies for bacterial wilt disease remain limited due to the lack of reliable chemical control methods and the pathogen’s environmental persistence. Chemical interventions frequently prove ineffective for eradicating soil-borne pathogens in infected fields, particularly in tomato cultivation. Therefore, the development of host resistance emerges as a promising approach, offering an environmentally sustainable and economically viable strategy for bacterial wilt management [10].

The most effective strategy for disease management is the development of disease-resistant varieties, which is widely recognized as the most economical and sustainable approach [11]. In the case of tomatoes, resistance to bacterial wilt is known to be a polygenic trait, involving the influence of multiple genes. Multiple resistant genes and Quantitative Trait Locus (QTL) mapping have been identified primarily through tomato cultivar ‘Hawaii 7996’ [12,13]. Major QTLs have been reported on chromosome 6 and 12, while minor QTLs were reported on chromosome 3, 4, and 8 [13,14]. Consequently, various genetic markers for trait screening have been successfully developed across multiple systems, such as the co-dominant SCAR markers [15] and SNP markers [16]. However, there are two key limitations regarding the use of these markers in Thai cultivars, which are the biological- and the technological-limitations.

The biological limitation emerges from the observation that the majority of infections in Thailand originate from distinct races and biovars of *R. solanacearum* compared to those employed in previous research. Primary infections in Thailand are predominantly caused by two strains: A3-25 (race 1, biovar 4) and Ka21 (race 1, biovar 3), whereas earlier investigations in tomatoes focused exclusively on race 1, biovar 3 [17,18]. Although previous studies have utilized QTL mapping with biovar 4 strains in chili pepper research [19], there remains a notable gap in comprehensive strain-specific studies. Consequently, potential divergence exists between the identified quantitative trait loci (QTLs) and their associated molecular markers when applied to different pathogen variants. To address this methodological discrepancy, conducting comprehensive disease assessment experiments utilizing the specific pathogen strains prevalent in Thailand is essential for generating accurate and regionally applicable results.

The technological limitation arises from performance deficiencies in QTLs derived from bi-parental populations, as demonstrated in previous studies [20]. Subsequently, the application of these markers in plant breeding programs has resulted in false positive outcomes, indicating that critical genetic information remains undetected [21]. To overcome these constraints, Genome-Wide Association Studies (GWAS) have gained widespread acceptance due to advances in Next-Generation Sequencing (NGS) technologies, which enable the generation of large quantities of sequence data at relatively low costs. GWAS enhances information coverage by utilizing the entire genome of available germplasm, which exhibits higher recombination rates, thereby facilitating the detection of genetic variations such as Single Nucleotide Polymorphisms (SNPs).

In this study, we employed the GWAS approach to identify genes and QTLs associated with resistance to bacterial wilt in Thai tomato cultivars. A total of 267 tomato accessions obtained from Thailand’s largest genetic collection, incorporating both local Thai varieties and international cultivars. A comprehensive set of 2.5 million SNPs was generated through whole-genome re-sequencing (WGrS) analysis. This SNP dataset was subsequently utilized to conduct GWAS for identifying SNPs associated with bacterial wilt resistance characteristics. For disease phenotyping, we specifically employed two predominant strains of *R. solanacearum*: A3-25 (race 1, biovar 4) isolated from Chiang Mai and Ka21 (race 1, biovar 3) obtained from Nong Khai, as these were the most common in major tomato-producing regions. A full list of *R. solanacearum* strains reported in Thailand is provided in Supplementary Table S1. Finally, SNP marker located on chromosome 12 was developed and successfully validated in F_2_ populations based on the identified associations.

## 2. Results

### 2.1. Estimation of Population Parameters and Genetic Differentiation Analysis

**Figure 1:**
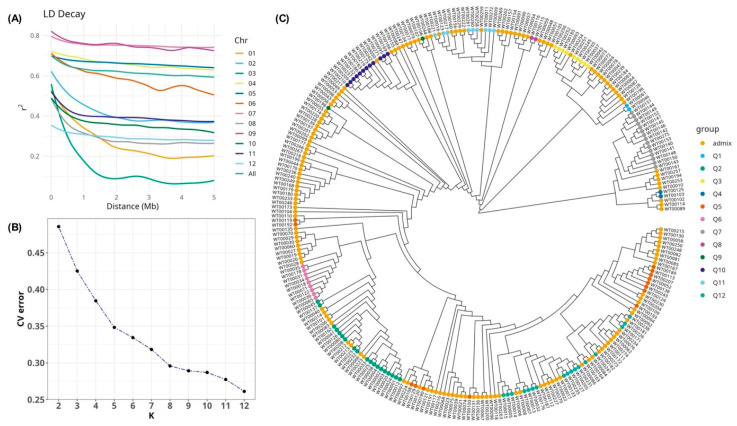
Estimation of population genetic parameters. (**A**) Average linkage disequilibrium (LD) decay across all 12 tomato chromosomes. The y-axis indicates the average pairwise correlation coefficient (r^2^), while the x-axis shows the physical distance between SNPs in megabases (Mb). Different chromosomes are distinguished by colored labels. (**B**) Population structure analysis based on cross-validation (CV) error plotted against the number of inferred subpopulations (K). (**C**) UPGMA dendrogram illustrating the genetic relationships among tomato accessions. Tip colors correspond to population structure groups inferred from ADMIXTURE (Q1–Q12 and admixture group).

The genetic structure of 267 tomato accessions was examined using multiple analytical approaches. Linkage disequilibrium (LD) exhibited a gradual decay, as indicated by relatively high r^2^ value (r^2^ > 0.2) extending beyond 5 Mb in most chromosomes, except for chromosomes 1 and 3, where LD decayed at approximately 3.3 Mb and 800 Kb, respectively (Figure 1A). Population structure analysis suggested an optimal number of genetic clusters at K = 12; however, the model selection metric continued to decline beyond this point, indicating the potential existence of additional substructures not fully captured at K = 12 (Figure 1B). Using a membership probability threshold of 0.8, the majority of accessions (n = 155) were classified as admixed, implying contributions from multiple ancestral populations without clear assignment to a single cluster. The remaining accessions were assigned to 12 discrete subpopulations (Q1–Q12), with Q2 being the most prominent (19 accessions), followed by Q12 (17), Q7 (15), Q5 (14), and Q10 (12). Smaller groups, including Q1, Q4, Q8, and Q9, each consisted of only 2 accessions, reflecting minimal representation (Figure 1C). Additionally, the SNP-based dendrogram revealed a nested hierarchical structure consistent with the admixture patterns, further supporting the inferred population structure (Figure 1C).

### 2.2. The Severity Assessment of Bacterial Wilt Disease

**Figure 2:**
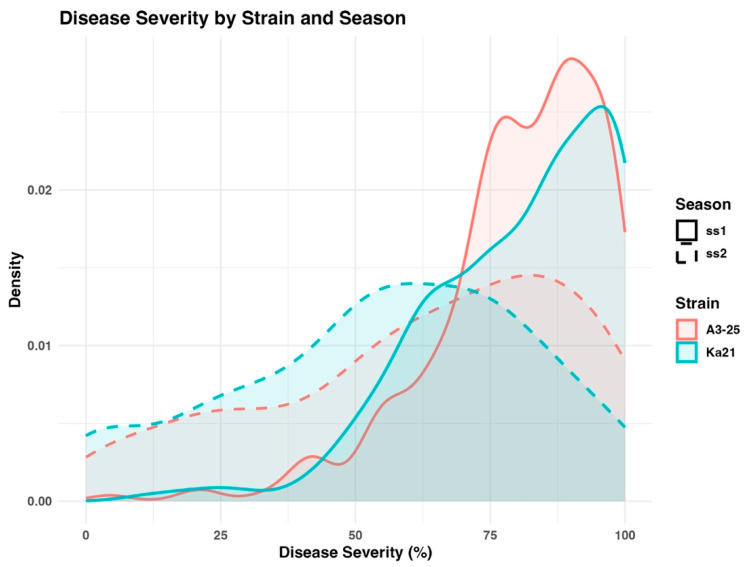
Distribution of disease severity (%) at week 5 post-inoculation for *R. solanacearum* strains A3-25 and Ka21 across the 2020 (ss1) and 2021 (ss2) experimental years. The density plot depicts the distribution of DSI values, highlighting phenotypic variation among the 267 tomato accessions evaluated and inter-annual variability between seasons.

Two strains of *R. Solanacearum* (*Rs.*) A3-25 and Ka21, were used to evaluate bacterial wilt resistance in a panel of 267 tomato accessions during two growing seasons, ss1 and ss2. Disease severity data collected at week 5 post-inoculation were selected for further analysis, as this timepoint represented the stage of maximum disease expression (Supplementary Table S2). The distribution of DSI values across the tomato panel is illustrated in the density plot presented in Figure 2. Both strains exhibited comparable disease patterns across experimental years, although minor variations were observed. The results demonstrate consistent and reproducible resistance responses among accessions, with decreased phenotypic variation observed in the susceptible classification groups in ss2.

The statistical analysis of the phenotypic assessment results is presented in Table 1. Strain A3-25 produced mean DSI values of 80.25 and 62.72 in 2020 (ss1) and 2021 (ss2), respectively, with a Pearson correlation coefficient of 0.32 and a broad-sense heritability estimate of 22%. Similarly, strain Ka21 exhibited mean disease severity indices (DSI) of 80.19 and 55.02 in 2020 (ss1) and 2021 (ss2), respectively, with a Pearson correlation coefficient of 0.48 between years and a broad-sense heritability estimate of 28%. A two-way ANOVA revealed significant main effects of Strain (F_1,3189_ = 13.61, *p* < 0.001) and Year (F_1,3189_ = 410.31, *p* < 0.001), as well as a significant Strain × Year interaction (F_1,3189_ = 13.07, *p* < 0.001), indicating that both the genetic background and environmental differences between years significantly influenced disease severity.

### 2.3. Genome-Wide Association Study (GWAS) for the Bacterial Wilt Resistant Trait

**Figure 3:**
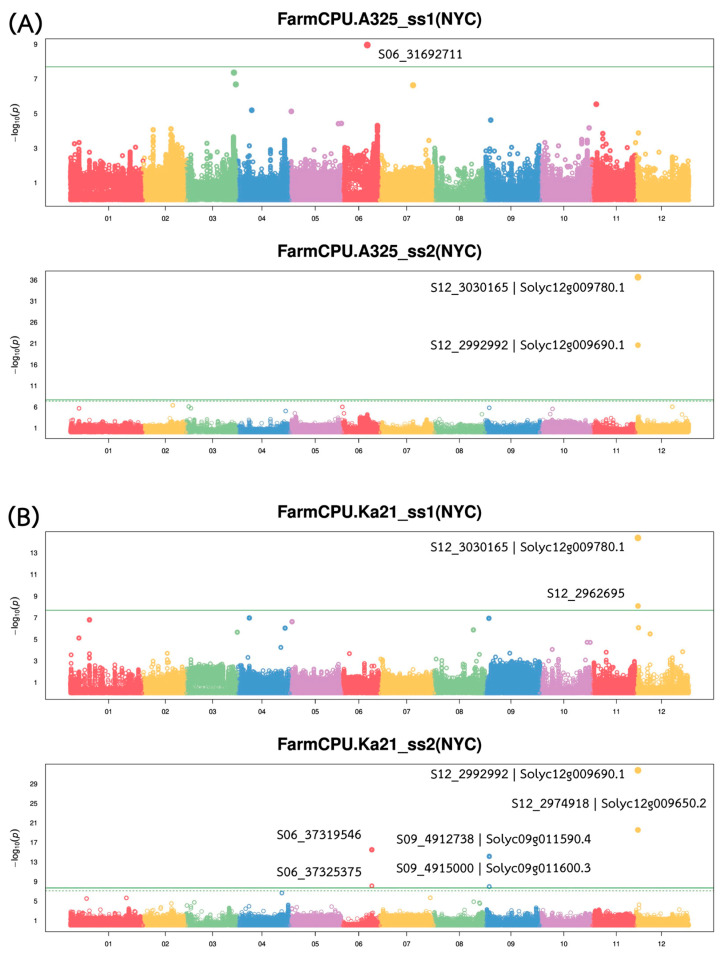
Manhattan genome-wide association study (GWAS) analysis of bacterial wilt resistance using the Fixed and random model Circulating Probability Unification (FarmCPU) approach. Results are shown for resistance against *R. solanacearum* (**A**) A3-25 and (**B**) Ka21.

Genome-wide association analysis revealed multiple quantitative trait loci (QTLs) associated with bacterial wilt resistance against both *Ralstonia solanacearum* (*Rs.*) strains, with major QTLs located on chromosome 12 (Figure 3). A comprehensive summary of GWAS findings is presented in Table 2. The strongest associations were detected within a ~70 Kb interval on chromosome 12 (Chr12:2962,695–3,030,165), where several SNPs showed high statistical significance and large effect sizes. Notably, SNP S12_3030165, annotated to *Solyc12g009780.1*, exhibited the most significant association with resistance to A3-25_ss2 (−log_10_(*p*) = 36.65 and R^2^ = 0.47), followed by SNP S12_2992992, linked to *Solyc12g009690.1* (−log_10_(*p*) = 20.60 and R^2^ = 0.47). These SNPs also demonstrated strong associations with resistance to Ka21_ss2, indicating a shared QTL contributing to resistance against multiple *Rs.* strains.

In addition to the major QTL on chromosome 12, moderate-effect loci were identified on chromosomes 6 and 9. On chromosome 6, S06_37319546 and S06_37325375 were significantly associated with Ka21_ss2, each explaining approximately 17–18% of the phenotypic variance. On chromosome 9, S09_4912738 and S09_4915000, annotated to *Solyc09g011590.4* and *Solyc09g011600.3*, respectively, were also linked to Ka21_ss2, with moderate significance (−log_10_(*p*) > 7.9) and effect sizes (R^2^ = 0.19–0.21). By contrast, a single SNP (S06_31692711) was weakly associated with resistance to A3-25_ss1, with a low explanatory power (R^2^ = 0.01), suggesting a minor or possibly spurious association. Overall, these findings indicate a polygenic basis for bacterial wilt resistance in tomato, with a major resistance QTL on chromosome 12 and additional moderate-effect loci contributing to resistance across other genomic regions.

### 2.4. Candidate Gene Mining

Reference genome analysis identified three candidate genes within this critical region (Chr12: 2993,595–3,030,165). These include *Solyc12g009650.2*, *Solyc12g009690.1* (receptor-like protein 12), and *Solyc12g009780.1* (receptor-like protein 6). *Solyc12g009650.2* encodes a proline-rich protein fragment that contains an InterPro domain (IPR013770) characteristic of plant lipid transfer protein (LTP) and hydrophobic helical proteins. LTPs are known to play roles in defense signaling and membrane dynamics. The other two candidate genes are leucine-rich repeat (LRR) receptor-like proteins, belonging to the receptor-like serine/threonine-protein kinase family, and are well-established components of plant innate immunity.

### 2.5. SNP Marker Development and Validation for Bacterial Wilt Resistance

Genome-wide association analysis identified a major locus on chromosome 12 associated with bacterial wilt resistance, where three candidate SNPs were located at positions 2,974,918 (CC/TT), 2,992,992 (CC/TT), and 3,030,165 (TT/AA). These SNPs were found within functionally annotated genes, specifically *Solyc12g009650.2*, *Solyc12g009690.1*, and *Solyc12g009780.1*, respectively. Haplotype analysis of these SNPs across 267 tomato accessions revealed seven distinct haplotype groups. The majority of accessions, comprising 213 individuals (79.8%), exhibited the homozygous reference genotype (CC/CC/TT). Thirty-six accessions (13.5%) carried the homozygous alternative genotype (TT/TT/AA), while 11 accessions (4.1%) displayed a heterozygous combination (CT/CT/TA). The remaining 7 accessions (2.6%) exhibited mixed allele patterns. This consistent allelic distribution highlights the informativeness and stability of the selected SNPs for genetic analysis. Among the three candidates, S12_2992992 was prioritized for marker development due to its location within a gene coding region, its strong statistical association with resistance trait (R^2^), and its consistent relevance to resistance against both *Rs.* strains A3-25 and Ka21 from the same growing season (ss2).

**Figure 4:**
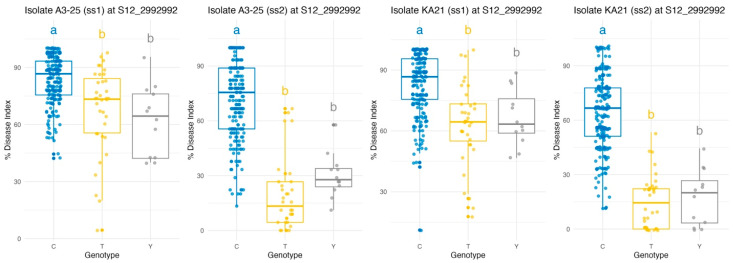
Genotype-wise variation in bacterial wilt resistance at SNP marker S12_2992992 across two *Rs.* isolates (A3-25 and KA21) and two seasonal replicates (ss1 and ss2). Boxplots show the distribution of disease index (%) across tomato genotypes carrying alleles C, T, and Y. Letters above the boxplots represent statistically significant groupings based on Fisher’s LSD test with Bonferroni correction (*p* < 0.05).

Haplotype-based analysis at the SNP locus S12_2992992 revealed significant genotypic variation in bacterial wilt resistance across both *Rs.* isolates (A3-25 and KA21) and two consecutive growing seasons (ss1 and ss2) (Figure 4). Across all four experimental conditions, analysis of variance (ANOVA) consistently demonstrated significant effects of genotype on disease response, with F-values ranging from 26.65 to 122.02 and highly significant *p*-values (*p* < 0.001), as shown in Table 3. Notably, genotypes carrying the reference allele C exhibited significantly greater susceptibility, reflected by higher disease index values, compared to those carrying the alternative alleles T or Y. In contrast, the T allele was repeatedly associated with the lowest disease index values, indicating enhanced resistance. Fisher’s Least Significant Difference (LSD) tests further substantiated these findings, consistently distinguishing the C allele from both T and Y alleles (*p* < 0.001 in all C vs. T and C vs. Y comparisons), while differences between T and Y were not statistically significant (*p* > 0.05), suggesting similar resistance levels between those genotypes. This pattern was robust across both strains and growing seasons, underscoring the stability of the association. These results highlight S12_2992992 as a reliable marker for bacterial wilt resistance in tomato, supporting its integration into marker-assisted selection programs. Moreover, this SNP represents a non-redundant, informative variant within the QTL region, facilitating precise and efficient marker development for breeding applications.

To validate the effectiveness of the selected marker, Kompetitive Allele Specific PCR (KASP) assays were conducted on an F_2_ population comprising 160 individuals derived from a cross between the resistant line ‘Hawaii 7996’ (TT) and the susceptible cultivar ‘Seedathip6’ (CC). The primer of the KASP marker is provided in Table 4.

**Figure 5:**
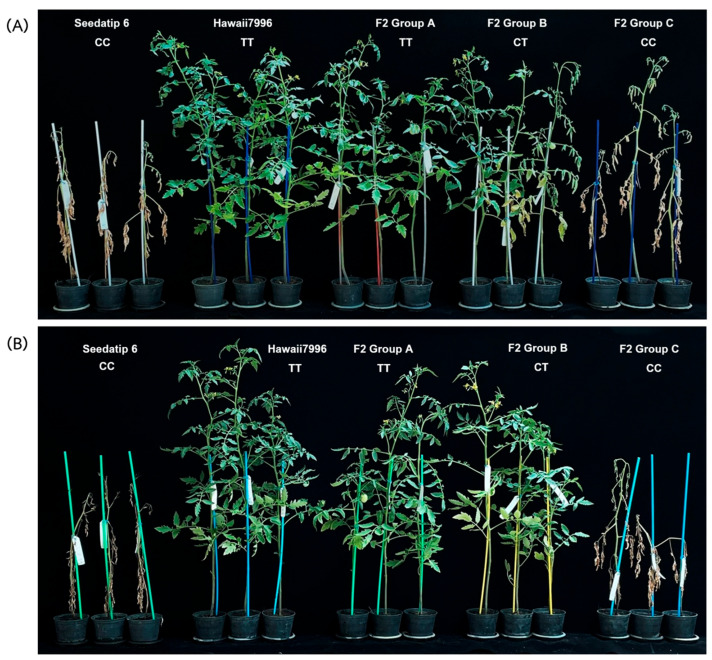
Evaluation of bacterial wilt resistance in the F_2_ population, categorized by genotype. (**A**) Inoculated with *Rs.* strain A3-25, and (**B**) inoculated with strain Ka21.

Both parental lines and the F_2_ progeny were evaluated under controlled inoculation conditions using both *Rs.* A3-25 (n = 82) and Ka21 (n = 78) (Figure 5). Disease severity varied widely among the F_2_ individuals, indicating clear segregation of resistance traits. Based on genotyping at the S12_2992992 locus, the population was classified into three genotypic groups: TT (n = 49), CT (n = 57), and CC (n = 54). The TT group, carrying the homozygous resistance allele, exhibited the lowest disease severity, with mean scores of 2.72 ± 2.49 for A3-25 and 1.50 ± 2.23 for Ka21. In contrast, the CC group, carrying the homozygous susceptible allele, showed higher disease severity, with mean scores of 4.50 ± 1.35 for A3-25 and 2.58 ± 2.34 for Ka21. The differences were statistically significant for A3-25 (*p* < 0.05), but not for Ka21. The heterozygous CT group displayed intermediate resistance, with severity scores of 4.10 ± 1.92 for A3-25 and 2.82 ± 2.50 for Ka21. These findings validate the association between the S12_2992992 marker and bacterial wilt resistance and demonstrate its potential utility in marker-assisted selection for breeding resistant tomato cultivars (Table 5).

## 3. Discussion

This study focused on the development of a molecular marker linked to bacterial wilt resistance in tomato. By integrating disease phenotyping with genome-wide association analysis and candidate gene identification, we aimed to generate a reliable tool to support marker-assisted selection. Upon examining the population structure, the analyses confirmed that the panel was highly diverse and admixed, revealing 12 genetic clusters among the 267 accessions, which indicates the presence of multiple subpopulations. High admixture between clusters was evident, reflecting the semi-domesticated origins of the panel. Moreover, linkage disequilibrium (LD) decayed very slowly across the genome, with pairwise r^2^ values remaining above ~0.2 even over several megabases. Such long-range LD is typical of cultivated tomato, relative to its wild relatives [22].

Disease severity was evaluated over two consecutive growing seasons, revealing consistent phenotypic differentiation between resistant and susceptible tomato accessions when challenged with two distinct *R. solanacearum* isolates, A3-25 and Ka21. Although annual environmental variation affected the absolute levels of disease severity, moderate heritability estimates (H^2^ ranging from 0.22 to 0.28) indicated that genetic factors play a substantial role in determining resistance. Correlation coefficients across trials further supported the presence of genetic control, validating the application of association mapping to dissect resistance traits [23]. These findings are in agreement with earlier reports of high broad-sense heritability for bacterial wilt incidence in tomato [24]. However, it is important to recognize that heritability estimates can be influenced by several factors, including the complexity of the trait, the environment, and the methods used for estimation. Among environmental factors, temperature plays a key role in disease development, with higher temperatures between 30 and 35 degrees Celsius known to increase plant susceptibility to infection [25,26].

Genome-wide association analysis using a mixed-model GWAS approach identified a major resistance locus on chromosome 12 that was significantly associated with bacterial wilt resistance in tomato. Several highly significant SNPs were concentrated within a narrow ~70kb interval corresponding to the major QTL (Bwr-12) interval. Notably, SNP S12_2992992 is located within the coding region of the gene *Solyc12g009690.1*, which falls within this major QTL interval and exhibited a strong statistical association with resistance, with an R^2^ value up to 0.47. Another significant SNP, S12_3030165, was also detected within this region. Thus, while the peak SNP resides within a candidate gene, the QTL refers to the broader genomic interval associated with the resistance trait, and this SNP likely represents the functional variant driving the QTL effect.

Bioinformatic analysis of this QTL interval revealed two genes, *Solyc12g009690* and *Solyc12g009780*, as putative resistance candidates. These genes encode leucine-rich repeat receptor-like proteins (LRR-RLPs), a well-characterized class of pattern recognition receptors (PRRs) that play a critical role in pathogen detection and immune response activation. To provide a more complete description, we have compiled the basic sequence characteristics of these candidate genes in Supplementary Table S3, including gene length, exon number, chromosome location, and functional annotation. LRR-RLPs, often functioning in concert with leucine-rich repeat receptor-like kinases (LRR-RLKs), act as cell surface receptors that recognize pathogen-associated molecular patterns (PAMPs) and initiate pattern-triggered immunity (PTI), a key plant defense mechanism [27,28,29]. In addition to the LRR-RLPs, the associated SNPs were located within or near genes encoding a proline-rich hybrid protein with a lipid-transfer domain. Both receptor-like proteins and hybrid proline-rich lipid-transfer proteins (HyPRPs) are well-documented components of plant immune signaling. Although transcriptome data for the accessions in this study are not available, literature reports indicate that LRR-RLPs and HyPRPs are typically upregulated in resistant tomato lines upon *R. solanacearum* infection, suggesting their likely involvement in defense responses. RLPs play central roles in basal defense responses [30], while HyPRPs have been linked to broad-spectrum disease resistance through their roles in cell wall reinforcement and signaling [31]. Collectively, the sequence information and literature-based evidence provide a theoretical basis for these candidate genes and support the rationale for developing robust molecular markers for bacterial wilt resistance in tomato. The co-localization of these immune-related genes with significant SNPs reinforces their potential involvement in conferring resistance to *R. solanacearum*. Taken together, these findings provide compelling molecular targets for functional validation and highlight the potential of using these markers in breeding programs aimed at enhancing bacterial wilt resistance in tomato.

The clustering of multiple strong association signals within this genomic region points to the presence of one or more key resistance genes. Notably, this region likely corresponds to the classical Bwr-12 quantitative trait locus (QTL) originally identified in the resistant cultivar ‘Hawaii 7996’. A functional SNP within *Solyc12g009690*, an LRR-RLP gene in this same region, was previously shown to perfectly differentiate resistant and susceptible lines [16]. Likewise, a SNP marker near Bwr-12 (RsR12-1) was developed and used in combination with a chromosome 6 marker to accurately distinguish resistant cultivars [32]. These findings reinforce the significance of this Chr12 locus as a reliable target for marker-assisted selection. While these efforts have significantly advanced marker-assisted breeding, they were often derived from bi-parental mapping populations or targeted resequencing approaches with limited genomic scope. In contrast, our study leveraged a diverse, admixed tomato panel and a genome-wide, hypothesis-free approach, which not only confirmed this resistance hotspot but also improved resolution and biological interpretability. The identification of the peak SNP S12_2992992 within *Solyc12g009690.1* provides a functional anchor within the major QTL interval, enhancing confidence in its relevance and translational value. Furthermore, the stability of this locus across pathogen strains and environmental conditions aligns with findings in other crops, such as peanut, where a major QTL (qBWB02.1) consistently explained a high proportion of resistance variation across years and settings [33]. The cross-strain detection and functional characterization of this Chr12 locus underscore its robustness and position it as a strong candidate for broad-spectrum resistance breeding.

Secondary resistance loci were mapped to chromosomes 6 and 9, findings that have been corroborated in other studies, thereby illustrating the polygenic architecture characteristic of bacterial wilt resistance [34,35]. Comparable results have been documented, where QTLs for bacterial wilt resistance in eggplants were also identified on chromosomes 3 and 6, with the QTLs on chromosome 6 overlapping with the BW-resistant QTL in tomatoes [36]. Additionally, candidate resistance loci on chromosome 3 in tomato have been identified, supplementing the known major loci on chromosomes 6 and 12, thus indicating that multiple loci contribute to resistance [18]. It was observed that the chromosome 6 region appeared to function synergistically with the major chromosome 12 locus, demonstrating additive resistance effects [13].

We examined the haplotype distribution of the top SNPs within the chromosome 12 resistance locus, including S12_2992992, across the diversity panel. Approximately 80% of accessions carried the reference haplotype, which is homozygous for the susceptible allele, while only about 13.5% possessed the alternate homozygous genotype associated with resistance. This skewed allele frequency highlights a strong opportunity for selection, as breeders could substantially enhance resistance levels by increasing the frequency of the minor, resistance-associated haplotype. S12_2992992 was prioritized for marker development due to its location within the coding sequence of *Solyc12g009690.1*, which resides within the major QTL interval, its clean segregation, and its strong statistical association with resistance phenotypes. Several markers have previously targeted the Bwr12 region, including the HRM-based KHU1 marker located at position 2,941,301 on chromosome 12 and its CAPS-converted version RsR121 [32]. However, subsequent evaluations showed that these markers, including one in *Solyc12g009690*, did not significantly outperform earlier tools [18]. In comparison, the KASP marker developed based on S12_2992992 provides higher resolution and accuracy than SCAR or CAPs/dCAPs markers, as it allows allele-specific detection via fluorescence and clear distinction between homozygous and heterozygous genotypes, reduces false positives, and is suitable for high-throughput applications (Supplementary Table S4).

The diagnostic utility of SNP S12_2992992 was validated in an F_2_ population derived from a cross between the susceptible cultivar ‘Seedathip6’ and the resistant ‘Hawaii 7996’. Genotypic analysis showed that individuals homozygous for the TT genotype, which corresponds to the resistance-associated allele, exhibited significantly lower disease severity than heterozygous (CT) or homozygous susceptible (CC) plants across both tested *Ralstonia solanacearum* strains. This strong genotype-to-phenotype correlation underscores the marker’s practical value in reliably distinguishing resistant from susceptible individuals, a critical trait for efficient breeding. Additionally, S12_2992992 is located within the coding region of *Solyc12g009690.1* and represents the peak SNP within the major QTL interval, suggesting both statistical significance and potential functional relevance. These findings build upon previous studies that employed CAPS and SSR markers to tag bacterial wilt resistance loci on chromosome 12 [18,32,37]. While earlier markers were linked to the Bwr12 locus, they showed limited improvement in selection efficiency. In contrast, the KASP-based S12_2992992 marker demonstrated stronger association signals, clearer segregation, and suitability for high-throughput, cost-effective screening, making it especially valuable for practical breeding applications.

## 4. Materials and Methods

### 4.1. Plant Materials, DNA Extraction and SNP Discovery

The plant material, DNA extraction, and SNP discovery procedures have been comprehensively described in our previous publication [22]. Briefly, a diverse collection of 267 tomato accessions was assembled from five distinct sources, encompassing Thai academic institutions, research organizations, and commercial varieties. The germplasm composition comprised 37.81% Thai (TH) accessions and 62.19% international (INT) accessions, representing genetic diversity from 20 countries worldwide.

Seeds were initially germinated on moistened tissue paper under dark conditions at room temperature. Following germination, seedlings were transplanted into peat moss-filled trays and subsequently transferred to individual pots containing a standardized growing medium consisting of loamy soil, perlite, coconut coir, and chicken manure in a 1:1:2:1 ratio. Plants were maintained under controlled greenhouse conditions throughout the experimental period.

Genomic DNA was extracted from young leaf tissue collected 1–2 weeks post-germination using the DNeasy Plant Mini Kit (Qiagen, Hilden, Germany) following the manufacturer’s protocol. DNA concentration was quantified using spectrophotometry, and only samples with concentrations exceeding 50 ng/µL were retained for subsequent analyses. DNA integrity and quality were verified through agarose gel electrophoresis to ensure high molecular weight DNA suitable for whole-genome sequencing.

Whole-genome sequencing was performed using Illumina HiSeq2500 and NovaSeq6000 platforms (Illumina Inc., San Diego, CA, USA). Raw sequencing reads underwent quality control and adapter trimming procedures. High-quality paired-end reads were aligned to the tomato reference genome SL4.0 using the Burrows-Wheeler Aligner (BWA) and processed with SAMtools v1.20 [38,39]. Single nucleotide polymorphisms (SNPs) were identified using the Genome Analysis Toolkit (GATK) HaplotypeCaller v 4.6.2.0 [40]. The resulting SNP dataset was subjected to stringent filtering criteria, including the removal of insertions/deletions (InDels) and multiallelic sites. Only biallelic SNPs with complete genotype data across all accessions and a minor allele frequency (MAF) greater than 5% were retained for downstream analyses [41].

### 4.2. Estimation of Population Parameters and Genetic Differentiation Analysis

Population parameters of 267 tomato accessions were analyzed. Linkage disequilibrium (LD) patterns were evaluated using PopLDdecay with a maximum distance threshold of 5 Mb [42]. Two binning intervals, bin1 = 100 and bin2 = 1000, were applied for LD decay visualization. Population structure was assessed using ADMIXTURE with K values ranging from 1 to 12 [43]. Cross-validation error was calculated to determine the optimal K. Variant Call Format (VCF) files were converted to FASTA format using VCF-kit v0.2.9 for phylogenetic analysis [44]. Pairwise genetic distances were computed in MEGA 11 using the Maximum Composite Likelihood method. Phylogenetic trees were constructed using the Unweighted Pair Group Method with Arithmetic Mean (UPGMA) algorithm [45].

### 4.3. Bacterial Strains and Plant Inoculation

Bacterial wilt resistance evaluation was conducted under controlled greenhouse conditions at the seedling stage. The assessment was performed in two independent trials to ensure reproducibility and robustness of results. The first trial was carried out from May to July 2020 (ss1), representing the rainy season, and the second from November 2020 to January 2021 (ss2), corresponding to the cool-dry season in Thailand. These time points were selected to account for potential environmental variation that may influence disease development. Two highly virulent *Ralstonia solanacearum* strains were used for inoculation: strain A3-25 (race 1, biovar 4), isolated from Chiang Mai, and strain Ka21 (race 1, biovar 3), isolated from Nong Khai. These strains were chosen to represent the predominant pathogen variants affecting tomato production in different regions of Thailand.

Bacterial inoculum preparation was conducted using modified Triphenyl Tetrazolium Chloride (TZC) medium. The base medium comprised Casamino acid-Peptone-Glucose (CPG) medium containing 5 g glucose, 10 g peptone, 1 g bacto casamino acids, and 1.5% agar per liter, supplemented with 50 mL/L of 2,3,5-triphenyl tetrazolium chloride solution. Bacterial cultures were initially incubated on TZC medium at 28 °C for 72 h, followed by transfer to fresh CPG medium for an additional 48 h. Subsequently, cultures were streak-plated on CPG medium and incubated for 24 h to ensure bacterial viability and purity. Bacterial cells were harvested and suspended in sterile distilled water, with cell density adjusted to an optical density of 0.3 at 600 nm (OD_600_ = 0.3) using a spectrophotometer, corresponding to approximately 1.0 × 10^8^ colony-forming units per milliliter (CFU/mL).

The inoculation procedure was performed on 4-week-old seedlings using a root-wounding technique. Each seedling received 10 mL of bacterial suspension (1.0 × 10^8^ CFU/mL) applied directly to wounded roots, with incisions made approximately 1 cm from the stem base to facilitate pathogen entry. A total of 35 seedlings per tomato accession were inoculated to ensure statistical reliability. The experimental design followed a Randomized Complete Block Design (RCBD) with seedlings systematically arranged in 3 blocks, each containing 3 replicate plants per accession [16,17].

### 4.4. The Severity Assessment of Bacterial Wilt Disease

**Figure 6:**
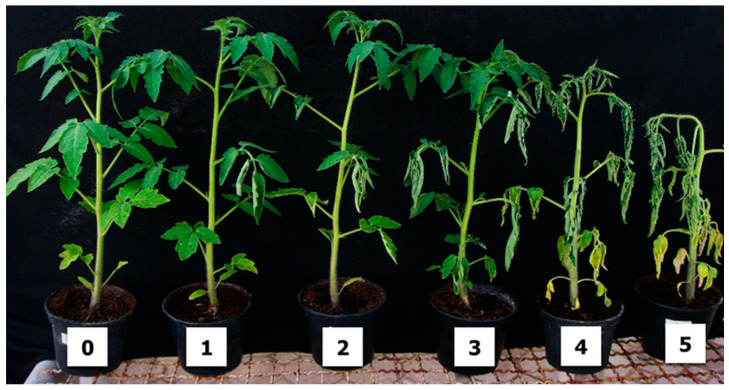
Disease severity scoring scale for bacterial wilt symptom assessment. Scores ranged from 0 (no visible wilting symptoms) to 5 (plant death), with intermediate scores representing: 1 = single wilted leaf; 2 = two to three wilted leaves; 3 = majority of leaves wilted with apical shoot remaining healthy; 4 = complete leaf wilting; and 5 = plant mortality.

Disease progression was systematically monitored at weekly intervals over a 5-week period following pathogen inoculation to capture the complete temporal dynamics of bacterial wilt development. Symptom manifestation and severity were evaluated using a standardized 6-point rating scale (0–5). The scoring criteria were defined as follows: score 0 indicated no visible wilting symptoms with plants maintaining healthy appearance; score 1 represented the initial symptom development with a single wilted leaf; score 2 denoted moderate symptom progression with two to three wilted leaves; score 3 indicated advanced disease development where the majority of leaves exhibited wilting while the apical shoot remained healthy; score 4 represented severe disease progression with complete leaf wilting but with the shoot still intact; and score 5 indicated plant death as the terminal disease outcome (Figure 6) [46,47].

Disease severity was quantified using the Disease Severity Index (DSI) calculated according to the described equation. The resulting DSI values were subsequently classified into distinct resistance categories based on predetermined criteria outlined in Table 6.

$$DSI\;\left(\%\right)=\frac{\sum \left(Class\;frequency\times score\;of\;rating\;class\right)}{\left(Total\;number\;of\;observations*maximal\;disease\;index\right)}\times 100$$

### 4.5. Genome-Wide Association Study (GWAS) and Candidate Genes Mining for Bacterial Wilt Resistant Trait

GWAS was conducted using GAPIT (8 March 2023) [48], employing the Fixed and Random Model Circulating Probability Unification (FarmCPU) approach. To account for population structure, the first three principal components (PCA) were included as covariates, while the kinship matrix was not explicitly included, as FarmCPU iteratively estimates fixed and random effects. To control for false positives, the Benjamini–Hochberg procedure was applied to adjust the *p*-values for multiple testing. In brief, *p*-values were ranked, and the adjusted significance values were calculated as

$$P_{adj}\left(i\right)=\frac{p\left(i\right)\cdot m}{i}$$

where *p*(i) is the i-th smallest *p*-value, i is its rank, and m is the total number of tests. SNPs were considered statistically significant at the genome-wide level if their FDR-adjusted *p*-values were below 0.05. Candidate genes associated with significant loci were identified based on the International Tomato Genome Sequencing Project reference genome SL4.0.

### 4.6. Development of KASP Primers and Genotyping

Sequences containing SNP of interest were submitted to assay design using Kompetitive Allele Specific PCR (KASP^TM^) genotyping assay (LGC Biosearch Technologies, Hoddesdon, UK). SNP-specific primers were designed using LGC’s proprietary PrimerPicker software. Each assay comprised two allele-specific forward primers and one common reverse primer to enable bi-allelic discrimination of target SNPs through competitive allele-specific PCR amplification. KASP genotyping reactions contained the SNP-specific assay mix, universal KASP Master Mix, and genomic DNA template. Thermal cycling was conducted according to the manufacturer’s protocol, followed by end-point fluorescence detection. Allele discrimination was achieved through competitive binding of allele-specific forward primers, each tagged with unique tail sequences complementary to universal fluorescence resonance energy transfer (FRET) cassettes labeled with FAM™ or HEX™ dyes (LGC Genomics, Teddington, UK). The KASP reaction procedure follows the Touchdown PCR reaction method. The specific steps include reaction at 95 °C for 5 min; reaction at 94 °C for 20 s, 61 °C for 1 min (10 cycles, each cycle temperature decreased by 0.8 °C); reaction at 94 °C for 20 s, 55 °C for 30 s, 72 °C for 1 min, a total of 26 cycles); reaction at 72 °C for 2 min. Amplification was performed using The Standard Thermo Scientific 384-well plate. The PCR products were scanned and analyzed by QuantStudio™ Real-Time PCR System (Thermo Fisher Scientific Inc., Waltham, MA, USA) under the fluorescence quantitative PCR instrument.

### 4.7. Statistical Analysis

All statistical analyses were conducted using R software version 4.2.1 [49]. Graphical representations were generated using the ggplot2 package version 3.5.2 [50], and dendrograms were created using the ggtree package version 3.12.0 [51]. Haplotype analysis was carried out using the geneHapR package version 1.2.4 [52]. Statistical analysis was performed using ANOVA from the stats package version 4.4.1 [49], and post hoc comparisons were carried out using the Least Significant Difference (LSD) test from the agricolae package version 1.3-7 [53]. In addition, chi-squared tests were performed on categorical resistance classifications (HR, R, MR, MS, S, and HS) to evaluate the association between SNP genotypes and bacterial wilt resistance levels.

## 5. Conclusions

This study successfully identified and validated a robust molecular marker, S12_2992992, that is strongly associated with bacterial wilt resistance in tomato. By integrating phenotypic evaluation, genome-wide association mapping, and candidate gene analysis, we highlighted a key resistance locus on chromosome 12, corresponding to the well-characterized Bwr-12 QTL. The resistance-associated allele of S12_2992992, located within a gene encoding an LRR receptor-like protein, was significantly correlated with reduced disease severity across diverse tomato accessions and confirmed in an F_2_ validation population. This SNP demonstrated stronger association and clearer phenotypic segregation than previously reported markers, underscoring its potential as a diagnostic tool for marker-assisted selection. The development of a KASP marker from S12_2992992 offers a practical, high-throughput approach to accelerate breeding of bacterial wilt-resistant cultivars. Importantly, although validated under Thai *R. solanacearum* strains, this marker targets a conserved resistance locus and can therefore be relevant for resistance breeding programs in other regions. These findings provide a valuable genetic resource for tomato improvement and reinforce the utility of integrating GWAS with functional genomics for trait dissection and crop enhancement.

## Figures and Tables

**Table 1:** The severity assessment of bacterial wilt disease.

Isolate	Year	Mean	Min	Max	SD	LSD (0.05)	Pearson’s Correlation	Heritability (%)
A3-25	2020 (ss1)	80.25	4.44	100	15.85	32.98	0.32	0.22
	2021 (ss2)	62.72	0	100	27.97	38.46		
KA21	2020 (ss1)	80.19	11.11	100	17.46	35.92	0.48	0.28
	2021 (ss2)	55.02	0	100	27.03	41.27		

**Table 2:** Significant SNPs identified associated with bacterial wilt resistance traits in tomato by GWAS.

SNP	Chr	Position	−log_10_(*p*)	Traits	Gene Annotation ^1^	R^2^	*p*-Value
S06_31692711	6	31,692,711	8.96	A3-25_ss1		0.01	1.01 × 10^−1^
S12_2992992	12	2,992,992	20.60	A3-25_ss2	*Solyc12g009690.1*	0.47	3.36 × 10^−38^
S12_3030165	12	3,030,165	36.65	A3-25_ss2	*Solyc12g009780.1*	0.47	6.38 × 10^−38^
S12_2962695	12	2,962,695	14.38	Ka21_ss1		0.19	5.72 × 10^−14^
S12_3030165	12	3,030,165	8.09	Ka21_ss1	*Solyc12g009780.1*	0.20	1.25 × 10^−14^
S06_37319546	6	37,319,546	15.51	Ka21_ss2		0.17	1.22 × 10^−12^
S06_37325375	6	37,325,375	8.13	Ka21_ss2		0.18	7.66 × 10^−13^
S09_4912738	9	4,912,738	14.16	Ka21_ss2	*Solyc09g011590.4*	0.19	4.80 × 10^−14^
S09_4915000	9	4,915,000	7.98	Ka21_ss2	*Solyc09g011600.3*	0.21	1.92 × 10^−15^
S12_2974918	12	2,974,918	19.57	Ka21_ss2	*Solyc12g009650.2*	0.44	3.40 × 10^−35^
S12_2992992	12	2,992,992	31.78	Ka21_ss2	*Solyc12g009690.1*	0.44	3.23 × 10^−35^

^1^ Gene annotation includes candidate genes within which SNPs are located. Sequence characteristics (gene length, exon–intron structure) are provided in Supplementary Table S3.

**Table 3:** Summary of ANOVA, Fisher’s LSD, and chi-square test results for genotypic effects on bacterial wilt resistance across *Rs.* strains and growing seasons.

Isolate	Years	ANOVA (F, *p*-Value)	Fisher’s LSD Grouping ^1^	LSD Pairwise *p*-Value	Chi-Square (X^2^, *p*-Value)
A3-25	2020 (ss1)	F = 26.65 *p* = 2.88 × 10^−11^	C > T = Y	C vs. T: 4.2 × 10^−8^ C vs. Y: 6.53 × 10^−6^ T vs. Y: 0.76	X^2^ = 66.20 *p* = 2.39 × 10^−10^
	2021 (ss2)	F = 122.02 *p* = 2.97 × 10^−38^	C > T = Y	C vs. T: 4.41 × 10^−35^ C vs. Y: 8.36 × 10^−11^ T vs. Y: 0.30	X^2^ = 190.84 *p* = 1.31 × 10^−35^
Ka21	2020 (ss1)	F = 29.71 *p* = 2.30 × 10^−12^	C > T = Y	C vs. T: 3.90 × 10^−11^ C vs. Y: 9.69 × 10^−4^ T vs. Y: 1	X^2^ = 56.14 *p* = 1.93 × 10^−8^
	2021 (ss2)	F = 115.67 *p* = 8.41 × 10^−37^	C > T = Y	C vs. T: 6.04 × 10^−32^ C vs. Y: 2.68 × 10^−13^ T vs. Y: 1	X^2^ = 164.12 *p* = 4.56 × 10^−30^

^1^ Fisher’s LSD Grouping: ‘>’ indicates statistically significant difference between groups (*p* < 0.05), while ‘=’ indicates no significant difference.

**Table 4:** Primer of KASP marker.

KASP Marker	SNPs	Primer Name	Sequence (5′-3′)
S12_2992992	T/C	FAM_primer	TGGTAATCAATTCGAAGGACCTGTCT
		VIC_primer	TGGTAATCAATTCGAAGGACCTGTCC
		Common R-primer	GACCAGCACAGTTGAGCAATGACAT

**Table 5:** Genetic analysis of 139 F_2_ individuals using the S12_2992992 marker.

Group	No. of Individuals	Mean of Disease ± SD		LSD Group ^1^	
		A3-25 (82)	Ka21 (78)	A3-25	Ka21
TT	49	2.72 ± 2.49	1.50 ± 2.23	b	a
CT	57	4.10 ± 1.92	2.82 ± 2.50	a	a
CC	54	4.50 ± 1.35	2.58 ± 2.34	a	a

^1^ Mean comparison were conducted using the Least Significant Difference (LSD) test. Means sharing the same letter are not significantly different at *p* < 0.05, based on the Bonferroni correction.

**Table 6:** Different resistance levels based on DSI.

DSI	Level of Resistance	Symbol
0–20	High resistance	HR
21–30	Resistance	R
31–40	Moderate resistance	MR
41–50	Moderately susceptible	MS
51–60	Susceptible	S
61–100	Highly susceptible	HS

## Data Availability

The datasets supporting the results and conclusions of this article are included within this article and its Supplementary Materials.

## Supplementary Materials

The following supporting information can be downloaded at: https://www.mdpi.com/article/10.3390/plants14193036/s1, Table S1: *Ralstonia solanacearum* strains from the Thai Department of Agriculture (DOA); Table S2: Disease assessment score and GWAS genotype; Table S3: Gene annotation; Table S4: A conceptual comparison of marker platforms.
